# The DNA damage induced by the Cytosine Deaminase APOBEC3A Leads to the production of ROS

**DOI:** 10.1038/s41598-019-40941-8

**Published:** 2019-03-18

**Authors:** Mathilde Niocel, Romain Appourchaux, Xuan-Nhi Nguyen, Mathilde Delpeuch, Andrea Cimarelli

**Affiliations:** CIRI, Centre International de Recherche en Infectiologie (CIRI), Univ Lyon, Inserm, U1111, Université Claude Bernard Lyon 1, CNRS, UMR5308, ENS de Lyon, 46 Allée d’Italie, Lyon, 69007 France

## Abstract

Human apolipoprotein B mRNA-editing catalytic polypeptide-like 3 proteins (APOBEC3s or A3s) are cytosine deaminases that protect cells by introducing promutagenic uraciles in invading retro-elements. However as a drawback of this protective activity, A3s can also target cellular DNA, leading to DNA damage and to the accumulation of somatic mutations that may contribute to tumorigenesis. Among A3s, A3A has been shown to be particularly proficient at mutagenizing cellular DNA, but whether this enzyme exerts additional effects on the cellular physiology remains unclear. Here, we show that A3A editing of cellular DNA leads to reactive oxygen species (ROS) production through Nox-enzymes. ROS production occurs in two distinct model cell lines and it is contingent upon DNA replication and intact enzymatic properties of A3A. For the first time, our results indicate that the editing activity of A3A results in the induction of a pro-inflammatory state that may possibly contribute to the constitution of a tumorigenic-prone environment.

## Introduction

Apolipoprotein B mRNA editing catalytic polypeptide-like 3 proteins (APOBEC3s, or A3s) are a family of cytosine deaminases composed of seven distinct members in humans (named A to H)^1^. A3s use preferentially single-stranded DNA as substrate of their enzymatic activity and catalyze the deamination of cytosines into uracils^2–6^. Cytosine deamination does occur spontaneously in cellular DNA, but in this case uracils accumulate at a much lower rate and are quickly disposed of by dedicated cellular enzymes^7,8^. In the case of invading retro-elements, A3s introduce a large number of mutations on the negative strand DNA that is then used as a template for the synthesis of the positive strand one during reverse transcription^2–5^. As a result, mutations become fixed on the viral genome as G to A transitions, ultimately leading to the element inactivation by mutagenesis^2–5,9–14^. In addition to this mechanism of inhibition, A3s has been also described to act through alternative mechanisms. Indeed, A3G is able to directly interfere with the process of reverse transcription through a cytosine-independent mechanism in the case of HIV-1^15–17^ and appears to inhibit indirectly Measles virus replication by modulating the activity of the mammalian target of rapamycin complex-1 (mTORC1)^18^.

A growing number of studies are revealing that as a drawback of what is a protective role of the cellular genome from invasion of *non-self* genetic elements, A3s expression may lead to the accumulation of somatic mutations^19–27^. These observations are of importance given that cancer genomic studies are unveiling the presence of an higher than expected accumulation of G to A transitions in nucleotide contexts evocative of A3s in cancer cells^19,28–37^. While these observations leave open the question of causality between editing and tumorigenesis, they clearly raise the possibility that cytosine deaminase enzymes may be involved either directly or indirectly in this process.

Among the members of the A3 family, A3A has received an increasing attention as a nuclear enzyme endowed with a proficient ability to deaminate not only foreign DNA introduced within the cell by transient transfection^38^, but also cellular DNA^21,25,26,39^. Expression of A3A induces a strong activation of several key mediators of the DNA damage response pathway, as the phosphorylation on Ser_139_ of the histone variant H2AX, the recruitment of 53BP1 and of the Replication Protein A (RPA) proteins and ectopic expression of A3A leads to cell cycle arrest and cell death^21,25,26,39^. Several studies have firmly linked these effects to the direct deamination of the cellular genome by A3A through its transient access to single-stranded DNA intermediates during cellular DNA replication^22,26^, followed by the action of Uracil-DNA glycosylases (UNG) and the recruitment of the apurinic/apyrimidinic (AP) endonuclease that create a site of lesion on the host genome. To add to the complexity of its action in cells, A3A appears regulated through multiple layers of control among which its nucleocytoplasmic distribution, or its interaction with cellular cofactors that influence its stability and enzymatic activity^40–42^.

In this work, we have used the controlled expression of A3A in two model cell lines (HeLa and U937, a cell line of myeloid origins) to explore the possible consequences of the expression of A3A in different cellular contexts. For the first time, we show here that the DNA damage induced by A3A leads to the production of reactive oxygen species (ROS) produced by NAD(P)H oxidases (or Noxes)^43,44^. We further determine that ROS production depends on the catalytic activity of A3A and that it is observed upon expression of both described A3A isoforms. These findings strongly support a previously proposed model^45^ in which contrarily to the well-described property of ROS to induce DNA damage, DNA damage may also initiate ROS production.

Given that ROS are well described inducers of DNA damage, we explored the possibility that they could exacerbate the extent of DNA damage already induced by A3A. Through the use of Nox inhibitors, we show that this is not the case, indicating either that the levels of ROS produced in this context is not sufficient to induce DNA damage, or that their effects is masked by the massive action of A3A. Contrarily to what observed in replicating cells, DNA damage as well as ROS production are not observed upon A3A induction in differentiated U937 cells, nor in dendritic cells (DCs) differentiated from primary monocytes and further stimulated with interferon alpha (IFNα), a strong inducer of A3A expression. Thus, these findings are in agreement with previous studies suggesting a strong requirement for cell cycle progression for the effects of A3A^22,26,27^ and suggest that both DNA damage and ROS production are unlikely to play a role in the antiviral properties of A3A, at least as it can be appreciated in non-dividing DCs. Rather, these findings suggest that these effects are a deleterious consequence of the mode of action of A3A in proliferating cells in which A3A not only directly mutagenizes the cellular genome, but also indirectly contributes to promote a proinflammatory, potentially pro-tumorigenic environment via ROS.

Overall, our study contributes to the understanding of the properties of A3A by highlighting for the first time that through a DNA damage response, A3A is likely to specify a more complex cellular program than previously anticipated.

## Results

### A working model to explore the functional consequences of A3A expression on the physiology of the cell

**Figure 1:**
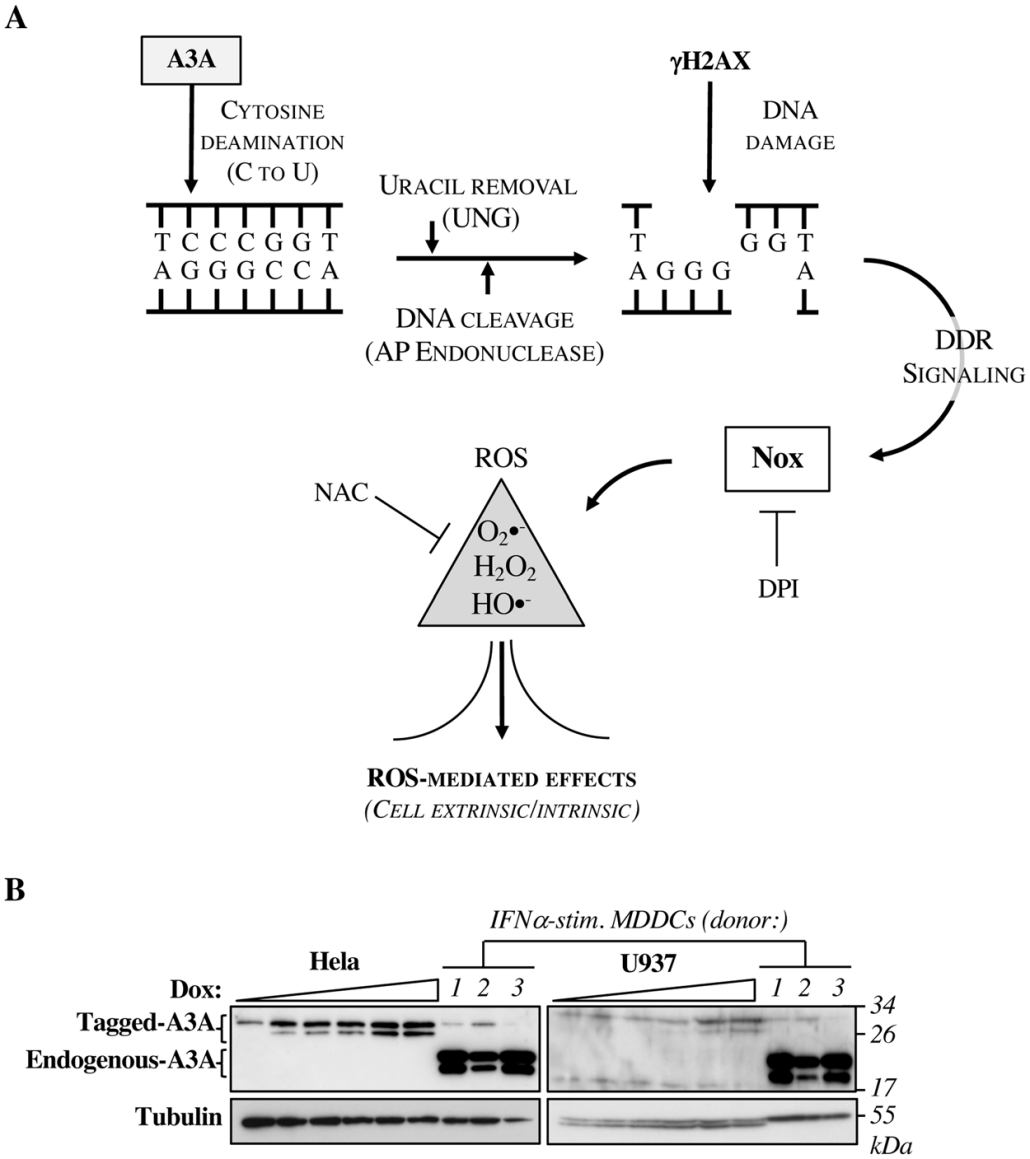
Schematic representation of the model tested in this study. (**A**) The cytosine deamination activity of A3A on genomic DNA leads to the accumulation of uraciles that upon removal by UNG lead to DNA cleavage by AP endonucleases and to a DNA damage that results in the recruitment of γH2AX on the DNA lesion. The ensuing DNA damage response (DDR) leads to activation of Nox enzymes that produce ROS. ROS can then exert multiple effects either intrinsic to the cell that produces them or extrinsic (inflammation, cell defense, cell death, DNA damage etc). Diphenyleneiodonium (DPI) is an inhibitor of Nox enzymes, while N-acetylcysteine (NAC) is an antioxidant that buffers ROS. (**B**) Stably-transduced cells expressing a dox-inducible form and HA tagged A3A were induced with increasing doses of doxycycline, prior to cell lysis and WB analysis forty-eight hours after induction. To compare these levels to those of endogenous A3A, primary monocyte-derived dendritic cells (MDDCs) stimulated for twenty-four hours with 1.000 U/mL of IFNα were also analyzed (three different donors displayed). The antibody used (Apo-C17) recognizes both A3G and the two isoforms of A3A which can be easily distinguished by their size^49^.

A3A induces massive editing of cellular DNA, resulting in the induction of a strong DNA damage response that leads to cell death. While solid experimental evidence supports these effects, the physiological consequences of the expression of A3A beyond DNA damage and cell death remain unclear. As schematically presented in Fig. 1A, we hypothesized here that the DNA damage response initiated by A3A could converge in the production of reactive oxygen species (ROS). While ROS are well known DNA damage inducers, a reciprocal link in which DNA damage leads to the production of ROS is less evident, although this possibility was clearly evoked in a past study in which ROS production was observed following the use of several DNA-damaging compounds (such as neocarzinostatin, doxorubicin, hydroxyurea^45^). ROS play numerous roles in the cell physiology beyond the induction of cell death, under both normal and pathological conditions^46^ and their production in the context of the A3A-induced DNA damage would offer a different perspective on A3A.

Retroviral-mediated gene transduction was used to engineer stable cell lines in which A3A expression was under the control of a doxycycline (dox)-dependent promoter. We selected in particular two model cell lines: HeLa cells that have been widely used in the past to explore the functional consequences of A3A expression^21,25,26,38,39^, and U937 cells, cells of myeloid origins that can be induced to differentiate into a non-dividing macrophage-like status upon PMA treatment^47^. To compare the expression levels of A3A in our dox-inducible cell lines with the ones observed in primary cells we used monocyte-derived dendritic cells (DCs, known to robustly express A3A upon IFNα stimulation) and the antibody ApoC17^48^. This antibody is a particularly useful tool as despite the fact that it was originally raised against A3G, it also recognizes A3A but not the other members of the A3 family (as we have extensively validated before^49^). Given that A3A and A3G bear distinct molecular weights (approximatively 21 and 42 kDa, respectively), this antibody allows an easy discrimination between these two proteins upon SDS-PAGE gel migration and WB. In our hands, U937 do not express detectable levels of A3A even after concomitant PHA and IFNα stimulation, (Supplementary Fig. 1). Upon dox-induction in our stable cell lines, the expression levels of A3A were comparable to those observed in primary DCs derived from different donors and stimulated with IFNα (Fig. 1B).

### A3A induces double strand breaks and ROS production in cycling HeLa and U937 cells, but not in PMA-differentiated U937 cells

**Figure 2:**
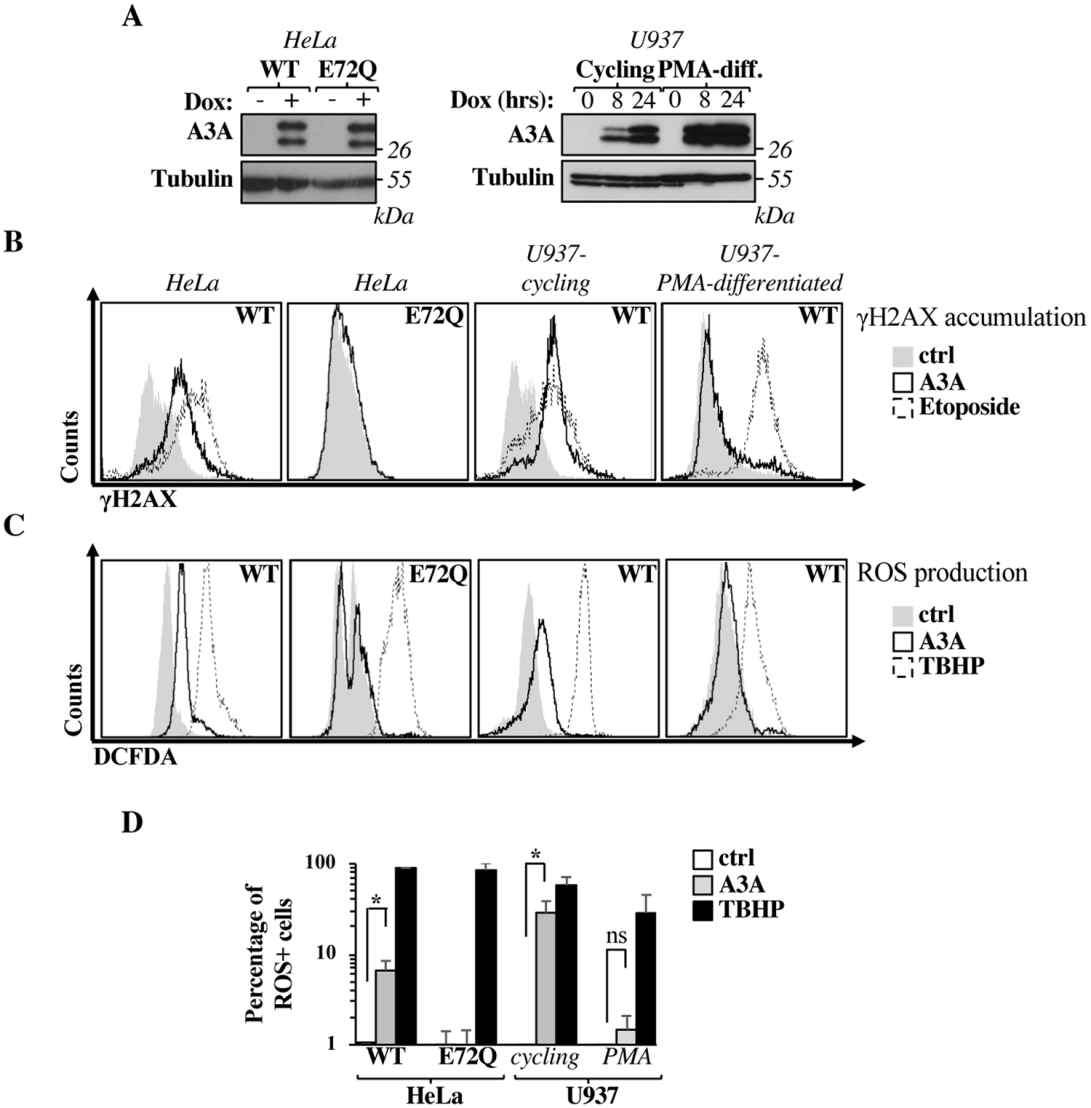
The expression of the catalytically active A3A enzyme leads to DNA and ROS production in cycling HeLa and U937 cells, but not in PMA-differentiated U937. (**A**) Stable cell lines expressing either wild-type (WT) or catalytically-inactive (E72Q) A3A were induced with doxycycline (1 and 0,5 µg/mL, in HeLa and U937, respectively) for twenty-four hours (or at an additional earlier time point, as indicated in the case of U937). Cells were then lysed for WB analysis using an anti-HA antibody directed against the HA epitope present on the C-terminus of A3A. Please note that: (1) translation of A3A results in the production of two isoforms, one of which smaller and generated by leaky scanning from an internal ATG start codon; (2) an additional band is recognized by the anti-Tubulin antibody in PMA-treated U937 cells, the identity of which is unknown. (**B**) The extent of accumulation of double strand breaks in the cell genome was assessed by intracellular staining and flow cytometry analysis of the phosphorylated form of the histone variant H2AX (on Ser 129, γH2AX) thirty-six hours after A3A induction. When indicated U937 cells were differentiated into a macrophage-like state upon incubation with PMA for two days, prior to doxycycline treatment. Etoposide was added as positive control (100 μM, 16 hours prior to analysis). (**C**,**D**) As in B but the production of ROS was measured by flow cytometry after incubation for four hours with DCFDA (20 μM). TBHP was added as a positive control. WB panels and histograms in (**A**–**C**) display representative results obtained. The graph in D presents mean ± SEM values corresponding to the percentage of ROS positive cells obtained from 3 (HeLa) to 10 (U937 cells) independent experiments. *p < 0.05 following a Student t test between the indicated conditions (two-tailed, unpaired; ns = not significant).

A3A expression was induced for twenty-four hours prior to WB analysis in HeLa as well as in U937 cell lines either cycling or growth-arrested upon incubation with PMA that induces their differentiation into macrophage-like cells (Fig. 2A). Given that preliminary experiments revealed an higher accumulation of A3A in U937 cells upon PMA-treatment when compared to untreated cells, A3A expression was detected at 24 and 8 hours post dox induction. The results obtained in this case indicate that the higher accumulation of A3A in PMA-treated U937 cells as opposed to cycling cells was observable at different time points. At present, whether this is due to higher translation rates or to decreased protein degradation’s rates remain unknown.

The extent of DNA damage was then measured by flow cytometry upon staining with an antibody specific for the phosphorylated form of the histone variant H2AX, a well described marker of DNA damage (Ser_139,_ γH2AX, as described in^39,25^, Fig. 2B). Under these conditions, substantial levels of γH2AX staining were observed in both cycling HeLa and U937 cells. The presence of DNA damage signatures was dependent on the intact enzymatic activity of A3A, given that none could be detected upon expression of a catalytically-inactive A3A mutant. On the contrary, no detectable levels of γH2AX staining were observed in differentiated U937 cells treated with PMA, indicating that cell differentiation prevented the accumulation of detectable levels of DNA damage upon A3A induction. These results essentially confirm previous studies^21,22,25,26,39,50^ and validate the experimental cellular models used in our study.

Next, we sought to determine whether the A3A-induced DNA damage response could converge in the production of ROS as measured by flow cytometry through the increase in fluorescence of cells incubated with the cell permeable dye 2′,7′–dichlorofluorescin diacetate (DCFDA). The fluorescence of DCFDA relies on oxidation and therefore DCFDA staining is a widely used technique to measure the accumulation of reactive oxygen species.

Under these conditions, a significant ROS production was observed in cycling HeLa and U937 cells (Fig. 2C for representative panels and 2D for cumulative data obtained in three independent experiments). The percentage of ROS-producing cells following the induction of A3A was lower than that obtained upon use of the positive control TBHP, yet it was consistent (Fig. 2C,D).

The production of ROS required the cytosine-deaminase activity of A3A, as no ROS were observed in cells expressing the catalytically-inactive A3A mutant (E72Q) and similarly, no ROS were produced in the absence of DNA damage in PMA-treated growth-arrested U937 cells.

Overall, these results indicate for the first time that A3A leads to ROS production in two distinct model cell lines and that this effect relies on the ability of A3A to attack cellular DNA through its cytosine deamination activity.

### ROS production occurs at similar levels for both described isoforms of A3A

**Figure 3:**
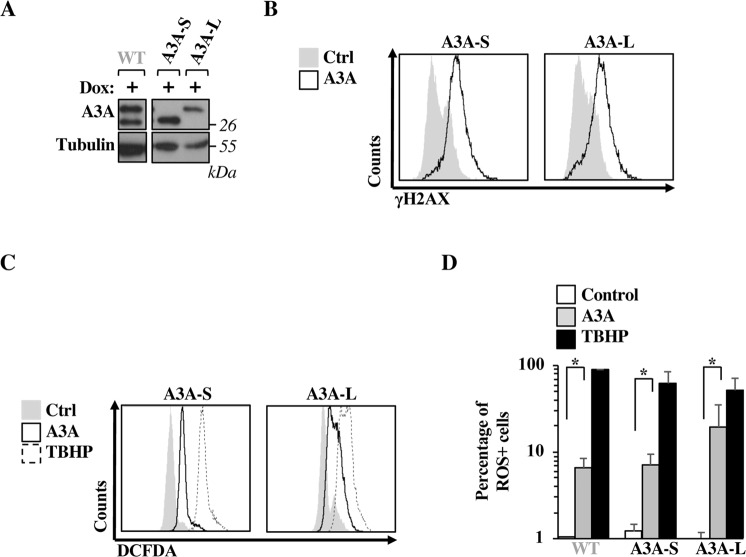
Both short and long A3A isoforms induce double strand breaks and production of ROS. (**A**) Short (A3A-S) and long A3A isoforms (A3A-L) were engineered by standard mutagenesis and stable HeLa cell lines were obtained as indicated in the legend to Fig. 2. The WB panels display typical expression profiles obtained and the migration of WT A3A is shown here solely for clarity’s sake (the same panel for WT A3A being presented in Fig. 2A). (**B**–**D**) After doxycycline induction (1 *μ*g/mL), cells were analyzed by flow cytometry to measure either the levels of double strand breaks, as well as ROS. The histograms present typical results obtained out of 3 independent experiments. The graph presents averages and SEM of three independent experiments. Results obtained with WT-A3A are simply reported here for comparison. A Student t test was performed between the indicated conditions (two-tailed, unpaired; *p < 0,05).

Translation of A3A from its own mRNA leads to the production of two protein isoforms: the full length and a shorter protein produced by leaky translation from an internal ATG and resulting in an N terminal deletion of twelve amino acids in A3A^51^, MEASPASGPRHL). Whether this shorter A3A isoform exhibits distinct behavior than the full length remains unknown and it was therefore important to determine if ROS production could be differentially mediated by one or the other isoform. To address this possibility, we engineered either the shorter A3A isoform (by removing the first twelve amino acids before the internal ATG, Δ12, designed here as A3A-S for short isoform) and the longer one (in which the internal Methionine was mutated to Isoleucine, preventing internal translation initiation, M13I, A3A-L, for long isoform, Fig. 3A, the WB for the WT A3A is provided here for comparison’s sake, as this lane is shown already in Fig. 2A). The expression of both short and long A3A isoforms in HeLa cells led to the induction of DNA damage (Fig. 3B), as well as to the production of ROS (Fig. 3C,D), indicating that under the conditions used here, no differences could be discerned between the two described isoforms of A3A in terms of induction of DNA damage and production of ROS.

### A3A-mediated ROS production occurs via a Nox-dependent mechanism

**Figure 4:**
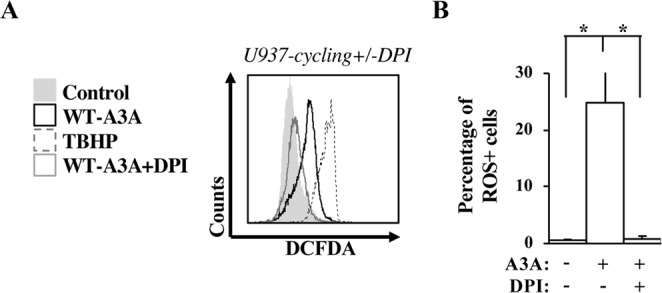
The A3A-mediated production of ROS occurs via Nox enzymes. (**A**,**B**) Cycling U937 were induced with doxycycline in the presence of the Nox inhibitor DPI provided at a concentration of 0,5 μM. ROS production was then measured 48 hours after the initial induction of A3A. A typical result is presented in the histogram of (**A**), while the quantification of the percentage of ROS-positive cells (Averages and SEM) obtained in four independent experiments is displayed in (**B**).

ROS are produced as a consequence of the reduction of oxygen by members of the nicotinamide adenine dinucleotide phosphate oxidases (NADPH oxidases, or Noxes, Nox 1 through 7). Members of this family share the presence of at least six trans-membrane helices, of a flavin adenine dinucleotide- and of a NAPDH-binding domain and display an heterogeneous pattern of tissular expression and of intracellular distribution^52^. To determine whether ROS production was dependent on Nox enzymes, diphenylene iodonium (DPI), a well-described broad Nox inhibitor was used in cycling U937 cells in which A3A expression had been induced. Under these conditions, DPI treatment of A3A-expressing cells completely abolished ROS production (Fig. 4A for a representative panel and 4B for a quantitative analysis of three independent experiments), indicating that the signals that originate as a. consequence of the DNA damage mediated by A3A converge on Nox enzymes leading to the production of ROS.

### The ROS produced in this context do not further exacerbate the extent of DNA damage caused by A3A

**Figure 5:**
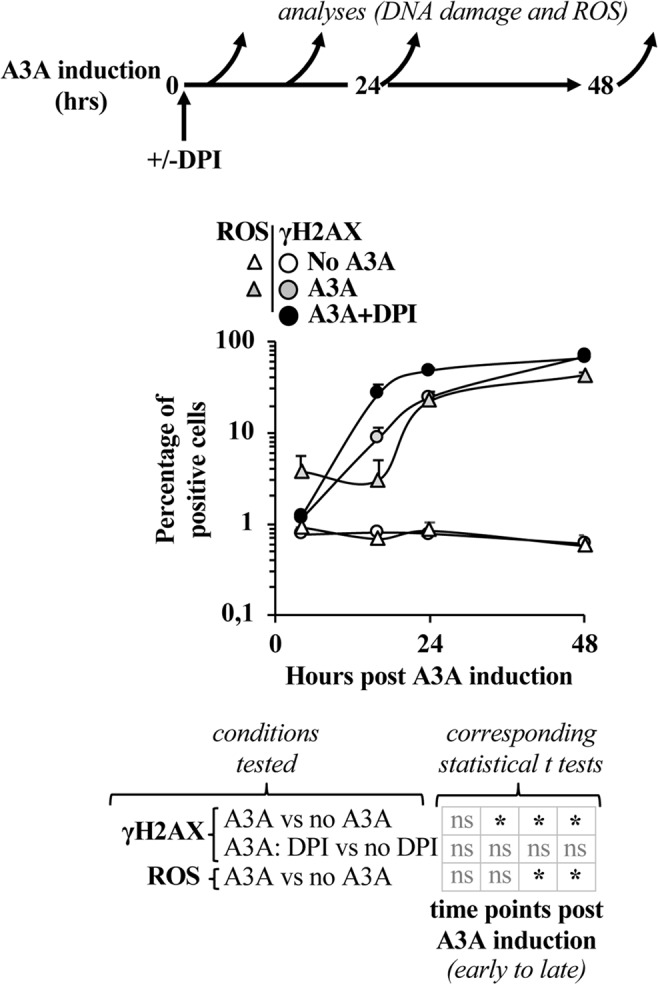
A3A-induced ROS production parallels DNA damage, but does not exacerbate it. For the time course analysis of the accumulation of γH2AX- and ROS-positive cells, cell aliquots were harvested at different times following the induction of A3A and then analyzed by FACS, as depicted in the scheme. When indicated, the Nox inhibitor DPI was added throughout the experiment. The graphs present averages and SEM obtained with three independent experiments. *p ≤ 0.05 and ns = non significant, following a Student t test between the indicated conditions (two-tailed, unpaired).

The results presented above support the notion that the activity of A3A is accompanied by the production of ROS. In turn, ROS are known to exert pleiotropic effects on the cell, among which the induction of DNA damage itself. To determine whether the ROS produced here upon A3A induction could contribute to -and perhaps amplify- the DNA damage response commonly attributed to A3A, a time curve analysis was performed in cycling U937 cells. To this end, cell aliquots were harvested at different times post A3A induction and the extent of DNA damage and ROS production were measured by FACS (Fig. 5). Under these conditions, the DNA damage marker used here started to be reliably detected by flow cytometry at 16 hours after A3A induction, increasing thereafter. This increase was paralleled by a similar accumulation of ROS-positive cells. Interestingly, a consistent proportion of ROS-producing cells became detectable already at 4 hours post A3A induction, at a time at which only basal levels of γH2AX-positive cells were present. We believe it likely that this rapid burst of ROS production in 3% of cells in which A3A expression has been induced may derive from a cell population in which DNA damage is ongoing, below the limits of detection of the flow cytometry-based assay used.

Importantly however, inhibition of Nox enzymes through the use of DPI failed to prevent the accumulation of γH2AX-positive cells indicating that, at least under the experimental conditions used here, ROS do not further contribute to the DNA damage induced by A3A.

### ROS production does not exacerbate the negative effects of A3A on cell survival

**Figure 6:**
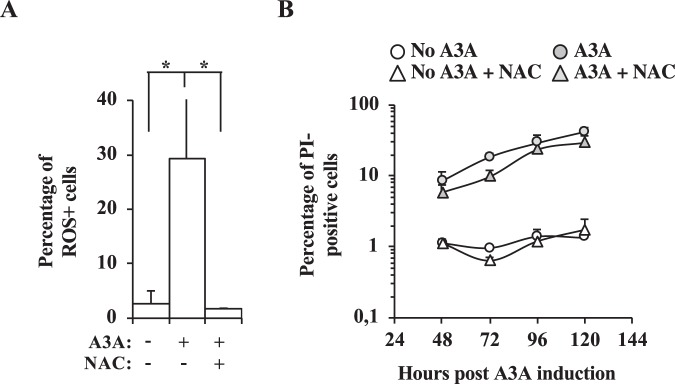
ROS induction by A3A does not aggravate the extent of cell death. Possible effects of ROS on the A3A-induced cell death were assessed by incubating cycling U937 cells treated or not with doxycycline with the ROS buffering compound NAC at a single time point to measure the effectiveness of ROS buffering by NAC (**A**) or at different time points after A3A induction (**B**). Cell death was measured after propidium iodide (PI) staining by flow cytometry. The graphs present averages and SEM obtained with three independent experiments. (**A**), *p ≤ 0.05 and ns = non significant, following a Student t test (two-tailed, unpaired). (**B**) no statistically significant differences were observed following the same test comparing plus versus minus NAC conditions.

A3A expression is commonly associated to cell death in cycling cells and given that ROS are also cytotoxic we set out to determine whether the A3A-dependent ROS production could contribute to the cytotoxic effects ascribed to A3A. To this end, we incubated cells with or without the ROS buffering agent NAC, that efficiently counters ROS production in cells (Fig. 6A), and we quantified the extent of cell death by propidium iodide staining and flow cytometry at different times post A3A induction (Fig. 6B). Under these conditions, A3A induction resulted in a substantial accumulation of PI-positive cells already at two days after induction and this percentage increased steadily over time. However, no significant reduction in the accumulation of PI-positive cells was observed in the presence of the ROS scavenger NAC. Overall, these results indicate that the ROS produced here do not further aggravate the extent of cell death already caused by A3A.

### The induction of A3A in primary DCs by IFNα stimulation does not lead to detectable DNA damage, nor ROS production

The results presented above as well as data in the literature indicate that cell cycling is a determining factor in the susceptibility of a given cell to the DNA damage and ROS production mediated by A3A. On the other hand, we have previously determined that A3A contributes to the modulation of HIV infection in myeloid cells and more specifically DCs and macrophages. Given that these cells act as central players of immune responses and that ROS have been described to be an integral part of antiviral responses, it was important to determine whether the expression of A3A in primary DCs could lead to a DNA damage response and to ROS that could in turn contribute to the poor susceptibility of these cells to viral infection or that could more generally play a role in the antiviral functions of DCs.

**Figure 7:**
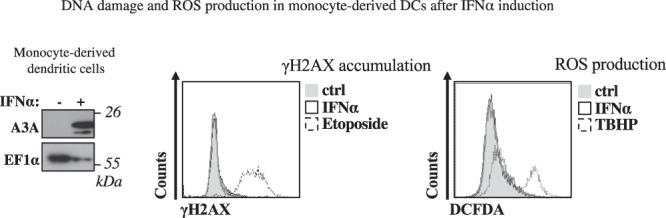
The induction of A3A in DCs via IFNα stimulation does not lead to DNA damage nor ROS production. Primary monocyte-derived dendritic cells (DCs) differentiated from monocytes of healthy donors upon incubation with GM-CSF and IL4 were stimulated for twenty-four hours with 1.000 U/mL of IFNα to induce the expression of A3A. Cells were then analyzed one day after by WB, or FACS to determine the extent of DNA damage and ROS production. One representative donor out of two is shown here.

As expected, treatment of DCs with IFNα strongly stimulated the expression of A3A (Fig. 7). However, IFNα stimulation and the ensuing increase in A3A did not lead to the accumulation of DNA damage signatures, nor to the production of ROS.

Overall, these results indicate that cell cycle progression is a general determinant in the susceptibility of target cells to both DNA damage and ROS production induced by A3A and suggest that these effects are unrelated to the antiviral functions played by A3A, at least in myeloid cells.

## Discussion

The major novelty of our study is to present evidence that the DNA damage response orchestrated by A3A culminates in the production of reactive oxygen species (ROS) through Nox enzymes, linking for the first time the mutagenic activity of A3A to a functional consequence outside the direct deamination of cellular DNA. This activity is observed in the two model cell lines tested and is dependent on active cell cycle progression, in line with the higher availability of single-stranded DNA substrates during DNA replication^19,22,26,28,39,40,53^. The strong requirement for cell cycle progression for the effects of A3A was also underlined by the fact that no DNA damage nor ROS were produced in either PMA-differentiated U937 cells, nor primary DCs, irrespectively of the manner in which A3A was induced (doxycycline or IFN, respectively). These results therefore indicate that the effects of A3A are heavily dependent on the cellular context in which this protein is expressed and the fact that myeloid cells (DCs, macrophages and monocytes) exhibit a non-cycling status is likely the main reason why these cells are protected from the deleterious effects of A3A.

While the presence of reactive oxygen species in the cell is well known to cause DNA damage, the possibility that DNA damage itself can drive the production of ROS has been evoked only in few studies^45,54^. In one of them, several DNA-damaging compounds were shown to drive ROS production via a Rac1-dependent mechanism that converged in the activation of Nox enzymes^45^. In line with this proposed mechanism of reverse control of ROS by DNA damage, we also show that a different DNA-damage agent, A3A, leads through cytosine deamination to the production of ROS by activation of Nox enzymes. In our hands, the levels of ROS generated via A3A do not exacerbate the extent of DNA damage already caused by A3A, nor its ability to cause cell death, implying that the direct deamination of the cellular genome by A3A plays a dominant function on these phenotypes and suggesting that ROS may act as second messengers, or mediate additional effects of A3A that remain to be discovered.

A number of well-conducted studies firmly established A3A as a potent mutagenic factor whose activity on genomic DNA results in extensive DNA damage in model cell lines^21–26,39^ and could in this manner contribute to tumorigenesis *in vivo*.

In addition to a direct contribution to this process through the introduction of somatic mutations in the cellular genome, our results indicate that A3A may also contribute to create an environment favorable to tumorigenesis through the induction of ROS. Indeed, ROS intervene in numerous cellular processes and clearly participate in chronic inflammation and have been clearly involved in the establishment of a pro-inflammatory, tumor-prone environment^55–65^.

To conclude, the results presented in this study indicate that A3A exerts far more complex effects on the cell physiology than previously appreciated and indicate that this enzyme bears the potential to favor the process of tumorigenesis through both direct and indirect mechanisms.

## Methods

### Plasmids, antibodies and cells

C-terminal HA-His tagged *wild-type* and mutant A3As were inserted in a pRetroXtight plasmid background (Clontech) by standard molecular biology techniques (WT, E72Q, ∆12 and M13I designed here as A3A-S and A3A-L). This vector DNA allows the generation of doxycycline-inducible, stably-transduced target cells upon retroviral-mediated gene delivery (details for the production of retroviral vectors are provided in the relevant section below). Antibodies used for WB were as follows: anti-apoC17 (that recognizes both A3A and A3G, easily distinguishable by size after SDS-PAGE gel migration as described in^49^, AIDS Reagents and Reference Program of the NIH, used in Figs 1B and 7 to compare/detect endogenous A3A with the A3A levels obtained in dox-inducible cell lines); anti-Tubulin and anti-HA (purchased from Sigma, T5168-2 and H3663, respectively; the latter of which used in the remaining figures to detect A3A in stable cell lines); anti-EF1α (purchased from Millipore, 05–235).

HeLa cells were maintained in complete DMEM media supplemented with 10% FCS, while the monocytic cell line U937 was propagated in complete RPMI 1640 media and 10% FCS.

To induce macrophage-like differentiation, U937 were treated with phorbol-12, myristate-13, acetate (PMA at 100 nM, SIGMA) for 2 days. Primary monocyte-derived dendritic cells were differentiated from blood monocytes of healthy donors upon incubation with 100 ng/ml of both granulocyte-macrophage colony-stimulating factor and interleukin 4 (GM-CSF and IL4, respectively, as described in^49^. Briefly, a fraction of enriched monocytes was obtained from peripheral blood mononuclear cells (PBMCs) after a Ficoll and then a Percoll gradient. The enriched monocyte fraction was then purified to homogeneity by negative depletion with a cocktail of antibodies-coated beads that removed contaminant cells (Miltenyi, Monocyte isolation kit II, 130-091-153, according to the manufacturer’s instructions). Cytokines were purchased from Eurobio (GM-CSF, 01-AR080; IL4, 01-A0050 and IFNα, PCYT-204). GM-CSF and IL4 were used at a final concentration of 100 ng/ml each for four days, while IFNα was used at 1.000 U/mL for twenty-four hours prior to analysis. Blood material consisted of discarded “leukopacks” obtained anonymously from the EFS-Lyon. Gender, race, and age of donors are unknown to the investigator as is the inclusion of women, minorities or children. This research is exempt from approval, although written informed consent was obtained from blood donors to allow use of their cells for research purposes.

### Retroviral vector production and generation of dox-inducible cell lines expressing A3A

Murine leukemia virus (MLV)-based retroviral vectors used here for gene transduction have been described before^66^. Briefly, retroviral vectors are produced by transient DNA transfection of HEK293T cells with 3 plasmids coding, respectively: the structural viral proteins Gag-Pro-Pol of MLV, the pantropic envelope glycoprotein G of the Vesicular Stomatitis Virus (VSVg) along with two miniviral genomes both pRetroX-based (ratio of 8:4:4:4, respectively). The first bears the A3A sequences under the control of a doxycycline-inducible promotor, the second codes for the rtTA transactivator (TetOn, Clontech). Virions released in the supernantant of transfected HEK293T cells were then purified through a 25% sucrose cushion, resuspended and their infection titers determined by exogenous-RT activity against standards of known infectivity. Cells were then transduced at a multiplicity of infection of 10 and stable cell lines were obtained upon selection with Puromycin and G418 present on the two different pRetroX constructs.

### Cellular assays

DNA damage was measured by flow cytometry with an Alexa Fluor 488-conjugated monoclonal antibody directed against the Ser139-phosphorylated form of the histone variant H2AX (γH2AX, 2577, Cell Signalling Technology). Unless otherwise specified, dox-treated cells were analyzed 36 to 48 hours after A3A induction. Cells were washed then fixed and permeabilized (Fix and Perm kit, GAS003, ThermoFisher Scientific, according to the manufacturer’s instructions) prior to incubation with the above-mentioned antibody. Etoposide was used as a positive control (E1383, used at 100 μM for 16 hours, Sigma). The extent of cell death was measured by propidium iodide staining by flow cytometry (PI, P3566, at 20 μg/mL, Invitrogen).

ROS production was performed using a cellular ROS detection assay kit, according to the manufacturer’s specifications (Abcam, ab 113851). Briefly, A3A expression was induced by incubation with dox and cells were then analyzed 36 to 48 hours post induction. Four hours prior to analysis, cells were counted and 10^5^ cells were incubated with 20 μM of the cell permeable fluorogenic dye 2′,7′–dichlorofluorescin diacetate (DCFDA) for 4 hours, prior to flow cytometry analysis. DCFDA is cleaved by cellular esterases within the cell, yielding a metabolized form that becomes fluorescent upon oxidation. The oxidative stress inducer, tertiary-butyl hydroperoxide (TBHP at 110 μM) was used as a positive control and for these analyses it was added 30 minutes after incubation with DCFDA for a total of 3.5 hours, prior to analysis. When specified the Nox inhibitor Diphenyleneiodonium (DPI, Sigma, 43088, used at 5 μM) and the ROS buffer N-acetylcysteine (NAC, Sigma, A7250, used at 7 mM) were added to the culture media at the time of induction of A3A.

In all cases, cells were analyzed on a FACSCalibur (BD Biosciences) using CellQuest Pro (BD Biosciences).

### Statistics

Student t tests were performed to determine statistically significance between the indicated conditions in the different experiments performed in this study, as specified in each figure legend.

## Supplementary information

Supp Figure 1, original blots

## Data Availability

All relevant data are within the paper.
