# Supplementary material for: The DNA damage induced by the Cytosine Deaminase APOBEC3A Leads to the production of ROS
Source: Sci Rep. 2019 Mar 18;9:4714. doi: 10.1038/s41598-019-40941-8 (PMC6423136; doi:10.1038/s41598-019-40941-8)

SUPPLEMENTAL MATERIAL

Supplementary Figure 1
and
Original blots for manuscript:

THE DNA DAMAGE INDUCED BY THE CYTOSINE DEAMINASE APOBEC3A LEADS TO THE PRODUCTION OF ROS

Mathilde Niocel[1], Romain Appourchaux[1], Xuan-Nhi Nguyen[1], Mathilde Delpeuch[1] and Andrea Cimarelli[1] §

[1] CIRI-International Center for Infectiology Research, Inserm, U1111, Université Claude Bernard Lyon 1, CNRS, UMR5308, Ecole Normale Supérieure de Lyon, Univ Lyon, Lyon, France

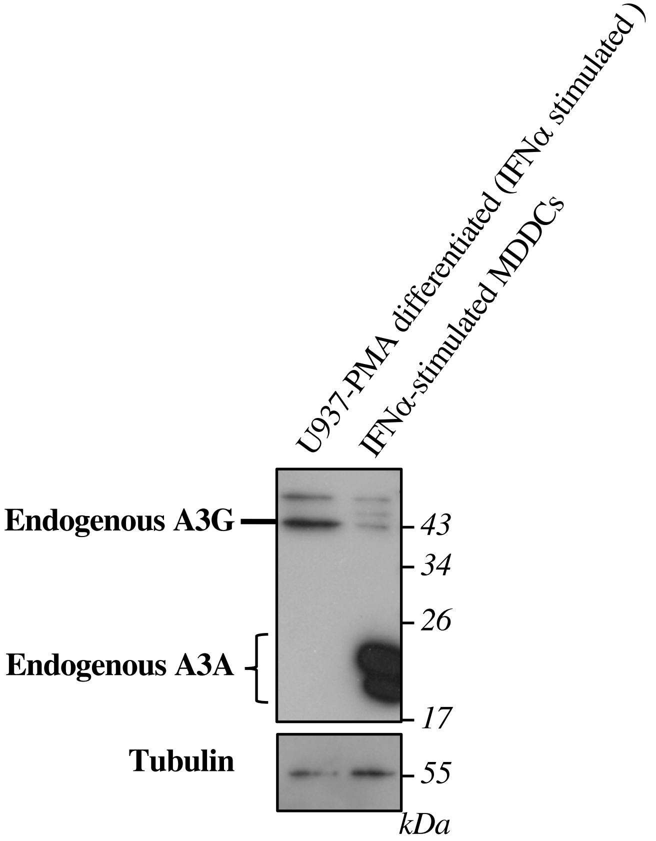

Supplementary Figure 1. A3A is not expressed in PMA-differentiated, IFNα-treated U937 cells. U937 cells were first differentiated into a macrophage-like status by a 24 hours treatment with PMA and then incubated for additional 24 hours with 1.000 U/mL of IFNα prior to cell lysis and WB analysis. Primary monocyte-derived dendritic cells (MDDCs) differentiated from monocytes of healthy donors upon incubation with GM-CSF and IL4 were similarly stimulated with IFNα and used here as controls in light of their well-described high expression of A3A. A representative donor is shown here. Please note that the antibody used (ApoC17 from the AIDS Reagents and Reference Program of the NIH) recognizes both A3G and A3A, easily distinguishable by their different size as we and others have already shown. The migration of endogenous A3A as a doublet has been well described in the literature. This doublet is due to initiation at two distinct ATGs, one of which is internal.

Figure 1B. Final figure

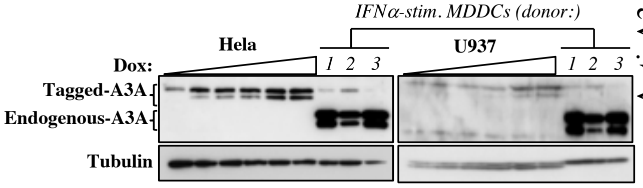

Corresponding blots

exposures of these blots overlaid with MW

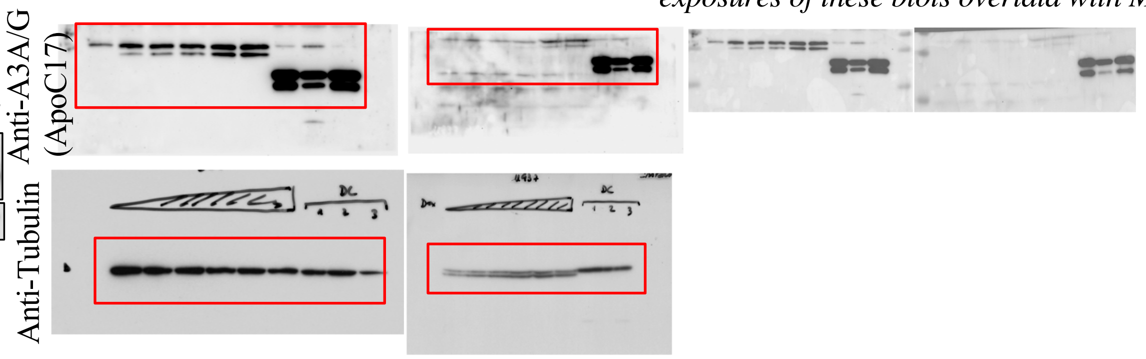

Figure 2A left panels. Final figure

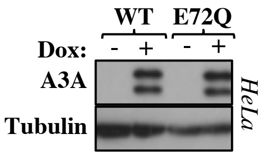

Corresponding blots

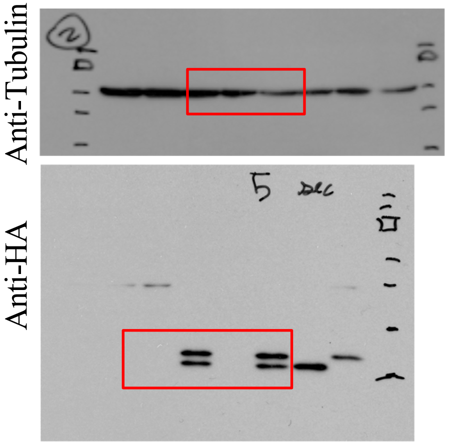

Figure 2A right panels. Final figure

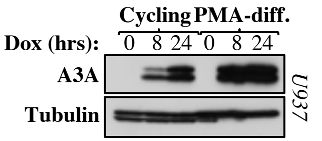

Corresponding blots

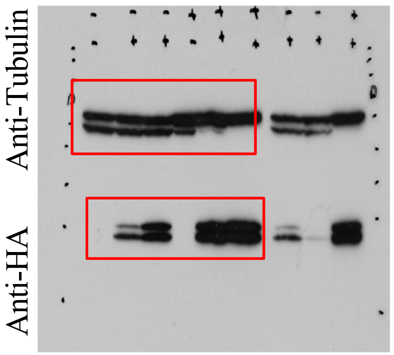

Figure 3A. Final figure

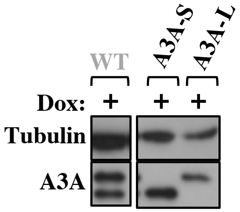

Corresponding blots

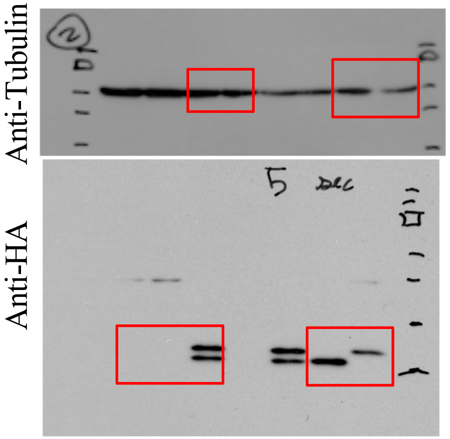

Figure 2A and 3A notice.

In the flow of the manuscript, we decided to describe separately the WT and E72Q mutant from the A3A-S and L isoforms although all were examined and are present on the same blots.
Also, as stated in the text, the WT panel in Fig 3A is the same of Fig 2A. It appears in grey and is presented simply to compare the isoforms migration pattern to the WT one.

FIGURE 7 FINAL FIGURE

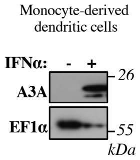

CORRESPONDING BLOTS

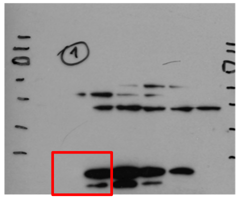

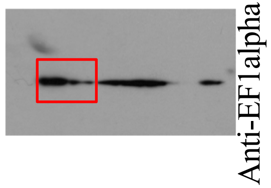

SUPPLEMENTARY FIGURE 1. FINAL FIGURE

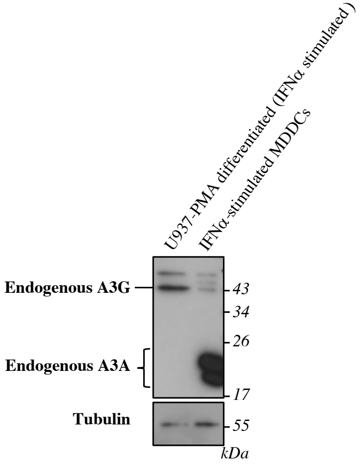

CORRESPONDING BLOTS

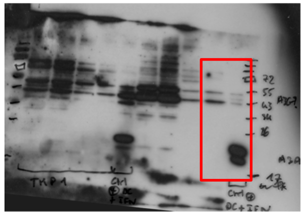

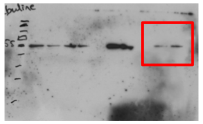

Supplement: Supplementary file 1 — Supp Figure 1, original blots [file 41598_2019_40941_MOESM1_ESM.pdf]
